# Unconscionable: how the U.S. Supreme Court’s jurisprudence lags behind the world when it comes to contraception and conscience

**DOI:** 10.1186/s40834-018-0055-z

**Published:** 2018-03-20

**Authors:** Aram A. Schvey, Claire Kim

**Affiliations:** 1 Previously Senior Policy Counsel and Manager of Special Projects at the Center for Reproductive Rights, Washington, DC, USA; 2 Fellow, Center for Reproductive Rights, Washington, DC, USA

**Keywords:** Conscience, Conscientious objection, Constitution, Contraception, First Amendment, Hobby Lobby, Law, Religion, Religious refusals, Supreme Court

## Abstract

U.S. Supreme Court jurisprudence undermines access to contraception by permitting individuals, institutions, and even corporations to claim religious objections to ensuring contraceptive insurance coverage, thus imposing those beliefs on non-adherents and jeopardizing their access to essential reproductive-health services. This jurisprudence is not only harmful but also runs contrary to the laws and policies of peer nations, as well as international human rights principles, which are more protective of the rights of health-care recipients to make their own decisions about contraception free from interference. The United States should look to the practice and jurisprudence of other nations and ensure that religious exemptions are not permitted to deprive a third party of access to contraception.

## Introduction

*“Your right to swing your arms ends just where the other [wo]man’s nose begins.”* 1

It is no surprise that in a religiously pluralistic society like the United States, there are a variety of beliefs about virtually every facet of life. Our diverse society is able to function because our secular government is supposed to safeguard individuals’ freedom to believe what they will, while simultaneously prohibiting them from imposing their beliefs on others. But when it comes to contraception, the U.S. Supreme Court has been inching towards a jurisprudence—that is, a body and philosophy of law—that allows some actors to impose their restrictive beliefs on others. This growing trend of permitting special religious exemptions that impede others’ access^2^ to contraception is not only dangerous but also represents a significant break from other developed, democratic countries.

Concern about religious refusals in the United States is particularly timely: On October 6, 2017, the Trump administration issued two interim final rules creating broad exemptions enabling employers, health-insurance providers, and universities claiming a religious or moral objection to contraception, allowing them to opt out of contraceptive coverage in their health plans, depriving beneficiaries—including employees, insurance holders, and students—of no-copay contraceptive coverage. Organizations—including universities—have flirted with the idea of eliminating no-copay contraceptive coverage, and others may follow suit.^3^ Although ongoing judicial review of the interim final rules has prevented their full implementation,^4^ thousands of women are at risk of losing access to affordable contraceptive coverage.

In January 2018, the U.S. Department of Health and Human Services (HHS) announced the formation of a new “Conscience and Religious Freedom Division” within the HHS Office for Civil Rights. It also proposed a new regulation that would vastly expand the potential scope of permissible religious objections to providing reproductive-health services. The proposed regulation would also forbid entities receiving federal funds from requiring any health-care entity to provide referrals for or even information about procedures to which the entity objects, and would threaten the withdrawal of federal funds from any state that requires contraceptive coverage.^5^ These expansions of religious and moral exemptions to the provision of health care are particularly dangerous in light of the Trump administration’s stated desire to reduce federal funding for family planning and contraceptive services, such as Medicaid and Title X, further limiting access to contraceptives for low-income and other women.^6^

The United States Constitution’s treatment of religion at first blush appears to be contradictory. On the one hand, the First Amendment restrains Congress from passing laws “respecting the establishment of religion,” while at the same time prohibiting it from passing laws “prohibiting the free exercise thereof.” These seemingly opposing dictates have resulted in incredible religious diversity and vibrancy, while at the same time ensuring a secular government.

Religious liberty, religious diversity, and secular government can coexist only because religious liberty is not a zero-sum game: One person’s religious liberty does not diminish someone else’s rights. Or as Thomas Jefferson eloquently stated, “The legitimate powers of government extend to such acts only as are injurious to others. But it does me no injury for my neighbor to say there are twenty gods, or no God. It neither picks my pocket nor breaks my leg.”^7^

In recent years, the U.S. Supreme Court has taken steps that call into question Jefferson’s understanding of religious liberty as something that is inherently harmless to others. Instead, in two cases involving contraception, the Court has suggested that one person’s religious liberty might permissibly come at the expense of another person’s right to access government-mandated no-copay reproductive health care. In *Burwell v. Hobby Lobby Stores*, the Supreme Court held that closely held for-profit corporations could hold and exercise religious beliefs and impose them on employees by refusing to comply with a nationwide no-copay contraceptive-coverage benefit.^8^ And in *Zubik v. Burwell*, the Supreme Court sidestepped issuing a decision on the merits in another challenge to the contraceptive-coverage benefit, failing to issue a strong ruling holding that a religious opt-out should not be permitted if it deprives others of essential reproductive-health services.^9^

The Supreme Court’s jurisprudence around contraception and conscience is not only misguided, but out of step with the preponderance of the policies and practices of other developed, democratic nations, which cabin religious opt-outs to ensure they do not result in harm to others. This Commentary highlights two principal areas in which the treatment of religious refusals in the United States differs from established international standards and the practices and policies of other democratic nations.

First, other democratic nations often limit the right to invoke religious refusals for certain health-care services to individuals directly involved in providing those services. Even in countries with conservative religious majorities, such as in Spain, Italy, and Colombia,^10^ the right to exercise a religious refusal is limited to direct health-care providers.^11^ The U.S. Supreme Court, however, has permitted those whose roles are attenuated from the objected-to service to interpose religious objections, including those who have no direct role in the provision of contraception or other health care, as well as institutions and for-profit corporations that offer health-insurance plans to their employees.

Second, recognizing that religious refusals in the context of health care can jeopardize the health and wellbeing of beneficiaries, many countries and international bodies explicitly limit religious objections and require adequate safeguards, such as ensuring informed consent and referrals to non-objecting providers, before a religious objection may be made. Sadly, the U.S. Supreme Court has neither acknowledged the government’s interest in ensuring women’s access to contraception,^12^ nor has it interpreted religious exemption laws to explicitly mandate protections even when women’s rights may be directly infringed by the objector’s refusal to provide some or all forms of contraceptives.

## Background

The Affordable Care Act profoundly altered the American healthcare landscape, in part by emphasizing preventive health services in a system that had heretofore been largely reactive. As part of the healthcare overhaul, the Affordable Care Act called for health-insurance plans to include coverage for preventive services without cost sharing for women. The list of preventive services for women was proposed by the Institute of Medicine and supported by the Health Resources and Services Administration. This list included, among other things, coverage for all U.S. Food and Drug Administration-approved contraceptive methods, sterilization procedures, and related patient education and counseling.

Since its implementation, the no-copay contraception benefit has become one of the most important and popular provisions of the Affordable Care Act, benefiting over 55 million women^13^ who no longer need to make a copayment for contraception (a year’s worth of oral contraceptives can cost upwards of $600, and long-acting reversible contraceptives, such as an intrauterine device or a contraceptive implant, can cost more than $1000—almost 1 month’s salary for a U.S. resident earning the federal minimum wage).^14^ ^15^ The implementation of the no-copay contraception benefit has thus resulted in a dramatic decrease in out-of-pocket medical costs for women.^16^ Studies show that decreases in cost-sharing has led to better adherence to and more consistent use of contraceptives, which has decreased the risk of unintended pregnancies.^17^

However, the newly minted contraceptive-coverage benefit was under attack almost immediately from conservatives, who sought to carve out religious exemptions to these coverage requirements, which initially applied to almost all employers other than houses of worship. The Obama administration sought to bridge the divide by proposing an “accommodation” for religiously affiliated non-profit organizations by allowing those employers to opt out of providing contraceptive coverage, which would instead be provided—at least in theory, seamlessly—by insurance companies. But this attempt at mollification only emboldened conservatives, who pushed to extend the accommodation to for-profit corporations.

In *Burwell v. Hobby Lobby Stores*, the Supreme Court held that closely held for-profit corporations are able to exercise religion, and ordered that the accommodation—which was intended for religiously affiliated non-profit organizations like Catholic hospitals—be made available to for-profit entities, including the craft-store retail giant, Hobby Lobby.

While the respondents in *Hobby Lobby* sought to avail themselves of the accommodation, the petitioners in *Zubik v. Burwell* sought to exempt themselves entirely, even objecting to filling out a one-page form notifying the government or their insurer of their objection. The petitioners in *Zubik* argued that doing so would make them complicit in the insurer’s provision of contraception. The Court failed to issue a substantive decision and took “no view on the merits” of the case; in so doing, it missed an opportunity to establish firmly the principle that religious objections cannot be interposed when they would harm the health of another person. Further, if U.S. courts allow the enforcement of the HHS October 6, 2017, interim final rules, any employer, health-insurance issuer, or university claiming a religious or moral exemption could choose to deny no-copay contraceptive coverage to women.

The United States’ unbalanced approach to religious and moral refusals is reflective of its position as a global outlier in its lack of protection for health rights. Globally, there is broad understanding that there is a human right to health. This is reflected in the International Covenant on Economic, Social, and Cultural Rights (ICESCR), a foundational international human rights treaty that recognizes the right of everyone “to the enjoyment of the highest attainable standard of physical and mental health.”^18^ The ICESCR has been ratified by 166 countries, but the United States is one of only few countries that have signed but not ratified the ICESCR.

The International Covenant on Civil and Political Rights (ICCPR), another foundational human rights treaty, which has been ratified by 169 countries, including the United States,^19^ states in Article 18 that everyone has the “right to freedom of thought, conscience[,] and religion.” However, the same Article *further* specifies that the freedom to manifest one’s religion and beliefs may be subject to limitations in order to protect the fundamental rights and freedoms of others: “Freedom to manifest one’s religion or beliefs may be subject only to such limitations as are prescribed by law and are necessary to protect public safety, order, health, or morals or the fundamental rights and freedoms of others.”^20^ While other countries have taken steps to balance religious and moral exemptions with the health and rights of those who may be affected by those exemptions, laws and jurisprudence in the United States are increasingly focused on privileging religious and moral exemptions of some at the expense of the health and safety of many others.

## Peer nations generally limit religious objections to individuals directly involved in health-care provision

The United States is an outlier in allowing corporations and groups with attenuated connections to the provision of contraception to raise religious refusals. First, peer^21^ countries tend to limit conscientious objections to individual human beings, and not institutions (and certainly not to for-profit corporations), based on the understanding that intimate, sincerely held beliefs are unique to individuals. And second, other countries tend to limit the right of religious refusals to physicians and those who *directly* assist in the objected-to service. To the contrary, the United States’ laws and jurisprudence allow a breathtakingly broad set of actors, including corporations that do not even provide health-care services or employ health-care providers, to raise religious refusals.

### Allowing corporations to interpose religious objections to contraception runs counter to the practice in peer countries

The U.S. Supreme Court in *Hobby Lobby* and *Zubik* has permitted institutions and even corporate entities to exercise religion and invoke religious refusals. In *Hobby Lobby*, the Court held that closely held for-profit corporations can further religious objectives and exercise religion within the meaning of the Religious Freedom Restoration Act.^22^ In *Zubik*, although the U.S. Supreme Court did not decide the case on its merits, it acknowledged that non-profit organizations, separate from individuals, may raise an argument for religious exemptions from laws.^23^

Other countries have expressly forbidden institutions and corporate entities from being able to claim exemptions. For example, the Colombian Constitutional Court has stated that “legal persons,” such as corporations, do not have a right to religious exemptions as they cannot experience “intimate and deeply-rooted convictions.”^24^ In another case, the Colombian Constitutional Court emphasized that conscientious objection is an “individual decision and not institutional or collective.”^25^

In Spain, the Spanish Bioethics Committee clarified that religious refusals only apply to individuals because “[o]nly individuals have conscience, not legal entities or other collective bodies.”^26^ Similarly, in Italy, only health-care personnel and their auxiliaries—such as nurses and assisting non-medical personnel—can invoke the right to conscientious objection, not institutions or hospitals.^27^ The French Constitutional Council has similarly noted that conscientious objections may be interposed by individuals rather than institutions when it upheld the repeal of Code of Public Health provisions allowing the heads of departments to object on behalf of their departments. Instead, the French Constitutional Council held that a department head’s individual right to object “cannot be exerted at the expense of that of other doctors and medical staff working in his service.”^28^ Still other countries, such as Denmark and New Zealand, only explicitly extend religious-refusal rights to individuals, and make no provision for institutional or corporate objections.^29^

The view that conscientious-objection rights inure only to individuals and not institutions and corporations has also been echoed at the global level by the Committee on the Elimination of Discrimination Against Women, which monitors the implementation of the Convention on the Elimination of all Forms of Discrimination Against Women. For example, in its concluding observations to Hungary in 2013, the Committee urged the country to ensure that conscientious objections by health professionals “remain a personal decision rather than an institutionalized practice.”^30^

The U.S. Supreme Court’s approach in *Hobby Lobby* is dangerous insofar as it permits corporations to object on behalf of all its employees, running roughshod over the consciences of individuals. As the dissenting opinion explains, such a broad understanding of corporate personhood invites corporations to seek religion-based exemptions from any regulations they may consider contrary to their “beliefs.”^31^

### Allowing those not involved in the provision of health care to object to its provision runs counter to the practice in peer countries

The Supreme Court’s decision in *Hobby Lobby* also runs counter to the practice in peer countries by allowing those not involved in the provision of health care, in this case contraceptive services, to object to its provision.

In addition to limiting religious refusals to individuals, most other countries limit religious refusals to physicians and other direct providers, and usually do not extend religious refusals to individuals only incidentally involved in the process. Those who are not direct providers are deemed by courts to be too attenuated in asserting a burden on their exercise of religion. For example, the United Kingdom has ruled in recent cases that although the Abortion Act of 1967 allows religious refusals for health-care professionals who “participate” in abortion, “participate” means “taking part in a ‘hands-on’ capacity”^32^ and thus a receptionist cannot claim a religious exemption from typing a letter referring a woman for a possible abortion.^33^

Numerous countries’ jurisprudence and legislation affirm such an understanding of who is eligible to invoke religious exemptions. Norway’s 1975 Abortion Act authorizing religious refusals explicitly states that it applies only to health personnel who either perform or assist in the operation itself.^34^ Similarly, in Spain, only health-care providers “directly involved” in the medical procedure may invoke a religious refusal.^35^ The Constitutional Court of Colombia similarly declared that “conscientious objection only applies to personnel that are directly involved in performing the medical procedure necessary to terminate the pregnancy.”^36^ In Italy, the law draws a further distinction between services that are specific to, and necessary for the interruption of pregnancy, and services that are merely incidental to it, and only persons providing services in the first category can invoke the right to conscientious objection.^37^

However, the U.S. Supreme Court in *Hobby Lobby* and *Zubik* allows *employers*—not just physicians or pharmacists, or even nurses and hospital administration staff—to invoke religious refusals against providing a health-insurance plan that includes certain contraceptive coverage. Despite the attenuation between providing a general health-insurance plan that covers contraceptives and the possibility of the destruction of an embryo through the independent actions of an employee who may use that contraceptive coverage, the United States’ highest court has refused to recognize any degree of attenuation. Instead, the Court declared that whether an action imposes a substantial burden on a petitioner’s religious freedom cannot be questioned by a court so long as the petitioner is sincere in believing that it does.^38^

The U.S. Supreme Court’s opinion starkly contrasts with the approach taken by many other high courts at peer democracies. In determining whether an objected-to action is too attenuated to warrant a religious exemption, many countries require an independent judicial finding about whether an act poses a burden on religious freedom instead of wholly accepting the petitioners’ argument that the act does.^39^ By making the test entirely subjective, anyone who feels that he or she is “complicit” in another person accessing contraception may have a legally cognizable claim, including those who are many steps removed.

## Peer nations ensure religious objections do not violate the rights of health-care beneficiaries

The United States is also a global outlier inasmuch as it requires few—if any—safeguards on the exercise of conscientious objection in the health-care setting. While other nations permit individual objections to the provision of health-care services, they recognize that religious-refusal rights must give way where their exercise would detrimentally affect another’s health. The European Court of Human Rights, for example, concluded that pharmacists who refused to sell contraceptives “cannot give precedence to their religious beliefs and impose them on others as justification for their refusal to sell such products.”^40^ However, instead of an approach that considers and protects the rights and freedoms of others to access certain health-care services, the United States has allowed the expansion of religious refusals to deprive women of their lawful access to no-copay contraceptive coverage.

Furthermore, other democracies consider access to reproductive-health services a fundamentally compelling interest that outweighs burdens on the religious freedom of providers and physicians. For example, in Finland, Iceland, and Sweden, concern for patients has led to policies that prohibit health-care providers from refusing based on religious beliefs to providing abortion services.^41^

Even when religious refusals are permitted in the context of sexual and reproductive health care, other democratic peer nations mandate robust patient-centered safeguards.^42^ Informed consent—a provider’s duty to inform the patient of all medically appropriate care, even when the provider may object to some of those medical procedures—is one such safeguard. The duty to refer a patient to a non-objecting provider is another.

### Peer nations require a patient’s informed consent before a religious exemption may be interposed

Numerous countries and international bodies have emphasized the importance of protecting a patient’s right to informed consent when religious refusals are granted. For example, the European Court of Human Rights has ruled that a Polish law violated the European Convention on Human Rights because it did not provide an effective mechanism for a woman to obtain diagnostic tests to determine fetal abnormality after her doctors’ refusal to conduct such tests on grounds of conscience.^43^ The Inter-American Commission on Human Rights similarly determined that “States must guarantee that women are not prevented from accessing information and reproductive health services [including contraception].”^44^ Countries have codified the duty of informed consent into statutes and guidelines, such as in South Africa, which requires physicians to provide objective, non-biased information on all available medical procedures.^45^

### Peer nations require that those interposing a religious objection to the provision of a health-care service provide a referral to a non-objecting provider

Another fundamental safeguard to protect women’s health is the duty of an objecting provider to refer a patient to a non-objecting provider. This duty is almost universally adopted in laws and medical ethical codes across Europe.^46^ For example, doctors in France who object to providing an abortion have a legal duty to refer the woman to another provider who is willing to perform the procedure.^47^ In the United Kingdom, the General Medical Council Guidelines specify that objecting doctors must inform patients of their right to see another doctor and make sure they have enough information to exercise that right.^48^

Beyond Europe, other regional and global human rights standards have affirmed and emphasized the duty to refer. The Inter-American Commission on Human Rights determined that in addition to the duty to ensure women’s access to information, “in situations involving conscientious objectors in the health arena, the States should establish referral procedures, as well as appropriate sanctions for failure to comply with their obligation.”^49^ And the Committee on the Elimination of Discrimination Against Women has stated in its General Recommendation No. 24 that “if health service providers refuse to perform [reproductive-health services for women] based on conscientious objection, measures should be introduced to ensure that women are referred to alternative health providers.”^50^

Medical associations have similarly emphasized the importance of referrals. International medical associations, such as the International Federation of Gynecology and Obstetrics (FIGO) and the World Medical Association, state in their code of ethics that practitioners who may have religious or moral objections to performing certain procedures must refer patients to practitioners who do not object or otherwise “ensur[e] the continuity of medical care by a qualified colleague.”^51^ In line with national and international practices, medical associations within the United States, including the American College of Obstetricians and Gynecologists and the American Medical Association, recognize a duty to refer in order to safeguard patients’ rights and access to certain reproductive health care.^52^

However, the United States has failed to mandate—and may even be undermining—such a duty. As noted above, the U.S. Department of Health and Human Services’ January 2018 proposed regulation would actually forbid entities receiving federal funds from requiring any healthcare entity to provide referrals for, or even information about, medical procedures they are unwilling to perform. And some states prohibit any medical institution that provides abortion care or referrals from participating in public-health programs or from receiving public funding of any sort.^53^ Some states even go so far as to deny tax-exempt status to any nonprofit hospital or health center that refers for abortion care.^54^

Legally mandated safeguards, such as the duty to refer, acknowledge that there are real negative impacts on the sexual and reproductive health of women who are denied services, and ensure that religious refusals do not impede women’s overall access. It is striking that these robust protections are lacking in the United States. Instead of national efforts to ensure that women’s health care is not compromised, proposed federal legislation such as the Conscience Protection Act, as well as existing state policies prohibiting certain referrals, seek to expand ways for institutions and individuals to deny care to women in the name of conscientious objection.^55^

## Conclusion

With respect to religious refusals affecting women’s access to contraception, the United States Supreme Court’s approach diverges from that of other democratic peer countries. First, the Court has embraced a startlingly broad understanding of who is eligible to invoke religious refusals. And second, the Court has emphasized the rights of those invoking religious refusals and de-emphasized the detrimental impact on the health and lives of women. The Supreme Court should look to the laws and practices of other democracies, which have more narrowly defined the scope of who can interpose a religious objection, and have mandated adequate protections to ensure that religiously motivated objections do not come at the expense of health-care recipients’ wellbeing.

## Footnotes

1 Chaffee Z. Freedom of Speech in Wartime, Harv. L. Rev. 1919;32:957 (bracketed material added to the original quotation).

2 The concept of “access” in the context of contraception encompasses both availability and affordability. Availability in this context means whether or not contraception can be obtained regardless of the price. For example, a pharmacist who refuses to dispense oral contraceptives to a woman, despite the fact that she has a valid prescription, undermines the availability of contraception. Affordability means that contraception is obtainable given the financial situation of the beneficiary. For example, even if every pharmacy and pharmacist were able and willing to dispense contraceptives, if those contraceptives were prohibitively expensive, women’s access to contraceptives would be severely limited even if they are nominally available. Within the United States, religious opt-outs implicate contraceptives’ availability *and* affordability. Health-care providers’ exercise of a legally permissible right to refuse to prescribe or dispense contraceptives limits their availability. And when employers—including for-profit corporations—refuse to include contraceptives as part of a health insurance plan, it can make contraceptives and related services unaffordable to many women.

3 For example, the University of Notre Dame announced in late October 2017 that it would end no-copay contraceptive coverage for employees and students by taking advantage of the religious exemption. The University of Notre Dame later reversed its decision in November 2017, citing “the plurality of religious and other convictions among its employees.” In November 2017, a spokeswoman for Georgetown University stated that the university is “reviewing the new [religious exemption] regulations.” Georgetown University later decided in December 2017 to continue its policy of providing no-copay contraceptive coverage via a third-party administrator. *See,* e.g., Cauterucci C. Notre Dame reverses its stance on birth control coverage for employees. Slate. 2017. http://www.slate.com/blogs/xx_factor/2017/11/07/notre_dame_reverses_its_stance_on_birth_control_coverage_for_employees.html. Accessed 31 Jan 2018; Hansen C. Amid uncertainty about insurance coverage, contraceptive access is already limited on religious campuses. U.S. News & World Report. 2017. https://www.usnews.com/news/national-news/articles/2017-11-09/amid-uncertainty-about-insurance-coverage-contraceptive-access-is-already-limited-on-religious-campuses. Accessed 31 Jan 2018; Ash E. Facing student presssure, georgetown continues contraception coverage in insurance plans. The Hoya. 2017. http://www.thehoya.com/facing-student-pressure-georgetown-continue-covering-contraception-health-insurance-plans/. Accessed 31 Jan 2018.

4 *See,* e.g., *California v. U.S. Dep’t of Health & Hum. Servs.*, No. 17-cv-05783 (D. Cal. Dec. 21, 2017) (order granting preliminary injunction); *Pennsylvania v. Trump*, No. 17-cv-04540-WB (D. Pa. Dec. 15, 2017), https://www.attorneygeneral.gov/uploadedFiles/MainSite/Content/Related_Content/PressReleases/BeetlestoneOrder.pdf (order granting preliminary injunction).

5 U.S. Dep’t of Health and Hum. Servs. Protecting Statutory Conscience Rights in Health Care; Delegations of Authority (RIN 0945-ZA03). 2018. https://www.federalregister.gov/documents/2018/01/26/2018-01226/protecting-statutory-conscience-rights-in-health-care-delegations-of-authority. Accessed 31 Jan 2018.

6 *See,* e.g., Nather D, Swan J. Deep spending cuts in Trump’s “taxpayer first” budget. Axios. 2017 https://www.axios.com/deep-spending-cuts-in-trumps-taxpayer-first-budget-1513302494-6e2e1532-98f1-461b-b0fd-938afb89b884.html. Accessed 31 Jan 2018; Beutler B. Leaked memo reveals white house wish list. Crooked Media. 2017. https://crooked.com/article/leaked-memo-reveals-white-house-wish-list/. Accessed 31 Jan 2018; Cunningham PW. The Health 202: Here’s the next way the Trump administration could go after abortion providers. Washington Post. 2017. https://www.washingtonpost.com/news/powerpost/paloma/the-health-202/2017/11/02/the-health-202-here-s-the-next-way-the-trump-administration-could-go-after-abortion-providers/59f9e9c230fb0468e765400d/?utm_term=.08d2003153f8. Accessed 31 Jan 2018.

7 Jefferson T. Notes on the State of Virginia. Boston: Lilly and Wait; 1832, p. 166, https://babel.hathitrust.org/cgi/pt?id=nyp.33433081883005;view=1up;seq=1*.*

8 *Burwell v. Hobby Lobby Stores, Inc.*, 134 S.Ct. 2751 (2014).

9 *Zubik v. Burwell*, 136 S.Ct. 1557 (2016).

10 Pew Research Center. Table: Religious Composition by Country, in Percentages. 2012. http://www.pewforum.org/2012/12/18/table-religious-composition-by-country-in-percentages/. Accessed 31 Jan 2018.

11 *See infra* Section titled “Allowing Those Not Involved in the Provision of Health Care to Object to Its Provision Runs Counter to the Practice in Peer Countries.”

12 In *Hobby Lobby*, the Court found it “unnecessary to adjudicate” the issue of whether there is a compelling government interest in promoting contraceptive access, deciding the case on other grounds. *Burwell v. Hobby Lobby Stores, Inc.*, 134 S.Ct. 2751 (2014).

13 U.S. Department of Health and Human Services. The Affordable Care Act is improving access to preventive services for millions of Americans. 2015. https://aspe.hhs.gov/pdf-report/affordable-care-act-improving-access-preventive-services-millions-americans. Accessed 31 Jan 2018.

14 *See,* e.g., National Women’s Law Center. The Affordable Care Act’s birth control benefit: too important to lose. 2017. https://nwlc.org/resources/the-affordable-care-acts-birth-control-benefit-too-important-to-lose/. Accessed 31 Jan 2018; Cleland C, Peipert J, Westhoff C, Spear S, Trussell J. Family planning as a cost-saving preventive health service. N Engl J Med 2011;364:e37; Center for American Progress. The high costs of birth control. 2012. https://cdn.americanprogress.org/wp-content/uploads/issues/2012/02/pdf/BC_costs.pdf. Accessed 31 Jan 2018.

15 Eisenberg et al. Cost as a barrier to long-acting reversible contraceptive (LARC) use in adolescents. J Adolescent Health 2013;52(4):S59-S63, http://www.jahonline.org/article/S1054-139X(13)00054-2/fulltext.

16 Becker NV, Polsky D. Women saw large decrease in out-of-pocket spending for contraceptives after ACA mandate removed cost sharing. Health Affairs 2015;34(7):1204–1211.

17 Henry J. Kaiser Family Foundation. The future of contraceptive coverage. 2016. https://www.kff.org/womens-health-policy/issue-brief/the-future-of-contraceptive-coverage/. Accessed 31 Jan 2018.

18 International Covenant on Economic, Social, and Cultural Rights, art. 12, Dec. 16, 1966. Similarly, the Constitution of the World Health Organization declares that “[t]he enjoyment of the highest attainable standard of health is one of the fundamental rights of every human being without distinction of race, religion, political belief, economic or social condition.” World Health Organization, Constitution of the World Health Organization, http://apps.who.int/gb/bd/PDF/bd47/EN/constitution-en.pdf?ua=1.

19 United Nations Treaty Collection, International Covenant on Civil and Political Rights, https://treaties.un.org/pages/ViewDetails.aspx?src=IND&mtdsg_no=IV-4&chapter=4&lang=en. Accessed 31 Jan 2018.

20 International Covenant on Civil and Political Rights, art. 18(3), Dec. 16, 1966.

21 The term “peer” in this context refers to other democratic, developed nations, such as the members of the Organisation for Economic Co-Operation and Development. *See* Organisation for Economic Co-operation and Development. Members and partners. http://www.oecd.org/about/membersandpartners/. Accessed 31 Jan 2018.

22 *Burwell v. Hobby Lobby Stores, Inc.*, 134 S. Ct. 2751, 2769 (2014) (“According to HHS [U.S. Department of Health and Human Services] and the dissent, these corporations are not protected by RFRA because they cannot exercise religion. Neither HHS nor the dissent, however, provides any persuasive explanation for this conclusion.”); *Id.* at 2771 (“If for-profit corporations may pursue such worthy objectives, there is no apparent reason why they may not further religious objectives as well.”).

23 *See Zubik v. Burwell*, 136 S. Ct. 1557, 1559 (2016) (“Given the gravity of the dispute and the substantial clarification and refinement in the positions of the parties, the parties on remand should be afforded an opportunity to arrive at an approach going forward that accommodates petitioners’ religious exercise while at the same time ensuring that women covered by petitioners’ health plans ‘receive full and equal health coverage, including contraceptive coverage.’”). The Court in this instance clarified that it was not ruling on the merits. *Id.* at 1560.

24 *See* Constitutional Court, May 28, 2009, Judgment T-388/09, 5.2, Gaceta de la Corte Constitutional (Colom.), http://www.corteconstitucional.gov.co/relatoria/2009/t-388-09.htm. Excerpts, O’Neill Institute Report on Conscientious Objection and Abortion, 25, 44 (2014), http://www.law.georgetown.edu/oneillinstitute/research/documents/WLWT-388-09English-FINAL.pdf.

25 Constitutional Court, Nov. 27, 2009, Judgment T-209/08 (Colom.).

26 *Comite de Bioetica de España*, Opinion of the Spanish Bioethics Committee on Conscientious Objection in Medical Care, p. 10 (2011) http://assets.comitedebioetica.es/files/documentacion/en/Conscientious%20objection%20in%20medical%20care_CBE_2011.pdf.

27 *See Legge 22 Maggio 1978*, n.194, art. 9, *Gazzeta Ufficiale 22 Maggio 1978*, n. 140 (Norme per la Tutela Sociale Della Maternità e sull’Interruzione Volontaria Della Gravidanza), *translation available at* http://www.columbia.edu/itc/history/degrazia/courseworks/legge_194.pdf; Republic of Italy, Ministry of Health, Report of the Ministry of Health on the Performance of the Law Containing Rules for the Social Care of Maternity and Voluntary Interruption of Pregnancy: 2014–2015, Dec. 7, 2016, at 44, http://www.salute.gov.it/imgs/C_17_pubblicazioni_2552_allegato.pdf.

28 Constitutional Court decision No. 2001-446 DC, June 27, 2001, Rec. 74, 11 (Fr.).

29 Sundhedsloven, LBK nr. 913 [Health Act, Law Notification no. 913], Ch. 28, § 102 (Den.), https://www.retsinformation.dk/forms/r0710.aspx?id=%20152710#Kap25; Contraception, Sterilisation, and Abortion Act 1977 § 46 (N.Z.), http://www.legislation.govt.nz/act/public/1977/0112/latest/whole.html?search=qs_act%40bill%40regulation%40deemedreg_abortion_resel_25_h&p=1#DLM18538.

30 CEDAW Committee, Concluding Observations: Hungary, para. 31, UN Doc. CEDAW/C/HUN/CO/7–8 (2013).

31 *Burwell v. Hobby Lobby Stores, Inc.*, 134 S. Ct. 2751, 2797 (2014) (Ginsburg, J., dissenting) (“[T]he Court’s expansive notion of corporate personhood—combined with its other errors in construing RFRA—invites for-profit entities to seek religion-based exemptions from regulations they deem offensive to their faith.”).

32 *Doogan and another v. Greater Glasgow and Clyde Health Board*, 38, UKSC 68 (2014), https://www.supremecourt.uk/decided-cases/docs/UKSC_2013_0124_Judgment.pdf.

33 *Janaway v. Salford Health Authority*, 3 All E.R. 1079 (H.L. 1988).

34 *Abortloven* [Abortion Act], 13 juni 1975 nr. 50, ch. II, § 20 (Nor.), http://www.ub.uio.no/ujur/ulovdata/lov-19750613-050-eng.pdf; Chavkin W, Swerdlow L, Fifield J. Regulation of Conscientious Objection to Abortion: An Internal Comparative Multiple-Case Study. Health Hum Rights. 2017;19(1):55–68. https://www.ncbi.nlm.nih.gov/pmc/articles/PMC5473038/.

35 Ley Orgánica 2/2010, de 3 de Marzo, de Salud Sexual y Reproductiva y de la Interrupción Voluntaria del Embarazo, BOE 2010, 3514, *translation available at* https://www.reproductiverights.org/sites/crr.civicactions.net/files/documents/Organic%20law%202-2010%20on%20SRH%20and%20voluntary%20pregnancy%20termination%20-%20English%20Translation.pdf.

36 Constitutional Court, May 28, 2009, Judgment T-388/09, 5.1, Gaceta de la Corte Constitutional (Colom.), http://www.corteconstitucional.gov.co/relatoria/2009/t-388-09.htm. Excerpts, O’Neill Institute Report on Conscientious Objection and Abortion, 25, 42 (2014), http://www.law.georgetown.edu/oneillinstitute/research/documents/WLWT-388-09English-FINAL.pdf.

37 *Legge 22 Maggio 1978*, n.194, art. 9, *Gazzeta Ufficiale 22 Maggio 1978*, n. 140 (Norme per la Tutela Sociale Della Maternità e sull’Interruzione Volontaria Della Gravidanza), *translation available at* http://www.columbia.edu/itc/history/degrazia/courseworks/legge_194.pdf;

38 *Burwell v. Hobby Lobby Stores, Inc.*, 134 S. Ct. 2751, 2778–79 (2014) (“The Hahns and Greens [Petitioners] believe that providing the coverage demanded by the HHS regulations is connected to the destruction of an embryo in a way that is sufficient to make it immoral for them to provide the coverage. This belief implicates a difficult and important question of religion and moral philosophy, namely, the circumstances under which it is wrong for a person to perform an act that is innocent in itself but that has the effect of enabling or facilitating the commission of an immoral act by another . . . [I]n these cases, the Hahns and Greens and their companies [Petitioners] sincerely believe that providing the insurance coverage demanded by the HHS regulations lies on the forbidden side of the line, and it is not for us to say that their religious beliefs are mistaken or insubstantial. Instead, our ‘narrow function . . . in this context is to determine’ whether the line drawn reflects ‘an honest conviction,’ and there is no dispute that it does.”).

39 *See,* e.g., *Janaway v. Salford Health Authority*, 3 All E.R. 1079 (H.L. 1988) (holding that a secretary’s act of signing an abortion referral letter did not constitute part of the treatment for abortion and thus was not covered by the conscience clause of the Abortion Act); *Legge 22 Maggio 1978*, n.194, art. 9, *Gazzeta Ufficiale 22 Maggio 1978*, n. 140 (Norme per la Tutela Sociale Della Maternità e sull’Interruzione Volontaria Della Gravidanza), *translation available at* http://www.columbia.edu/itc/history/degrazia/courseworks/legge_194.pdf (stating that health-care personnel providing pre- and post-abortion care cannot claim religious exemption); Zampas C, Andión-Ibañez X. Conscientious Objection to Sexual and Reproductive Health Services: International Human Rights Standards and European Law and Practice, Eur. J. Health L. 2013;19:247 (citing Regulations for the Implementation of the Act dated 13 June 1995 no. 50 concerning Termination of Pregnancy, with Amendments in the Act dated 16 June 1978, no. 66, § 20 (Nor.)) (stating that the right to refuse to assist in an abortion does not apply to staff providing care or treatment to the woman before or after the procedure); *Id.* at 250 (citing Auto del Juzgado Contencioso-Administrativo No. 3 de Malaga, Pieza separada medidas provisionales no. 12.1/2011, Pmto. Especial proteccion derechos fundamentals no. 39/2011 (Mar. 29, 2011) (Spanish court holding that a family doctor at a public medical center could not object to give referrals to women seeking abortions).

40 *Pichon and Sajous v. France*, No. 49853/99, Eur. Ct. H.R. (2001) (inadmissible) (holding that pharmacy owners’ claim that they could refuse to sell contraceptives based on their religious beliefs is inadmissible under the European Convention on Human Rights because pharmacy owners cannot prioritize their beliefs over their professional obligations).

41 Heino et al. Conscientious Objection and Induced Abortion in Europe, Euro. J. Contracept. Reprod. Health Care 2013;18:231-33; Fiala C, Arthur J. There Is No Defence for ‘Conscientious Objection’ in Reproductive Health Care, Eur. J. Obstetrics & Gynecology & Reprod. Bio. 2017;216:254-256.

42 Zampas C, Andión-Ibañez X. Conscientious Objection to Sexual and Reproductive Health Services: International Human Rights Standards and European Law and Practice, Eur. J. Health L. 2013;19:252–53.

43 *R.R. v. Poland*, No. 27617/04, Eur. Ct. H.R. (2011).

44 IACHR, Access to Information on Reproductive Health from a Human Rights Perspective, 94–95, 99 (2011), http://www.cidh.oas.org/pdf%20files/womenaccessinformationreproductivehealth.pdf.

45 *See* Health Professions Council of South Africa, General Ethical Guidelines for Reproductive Health, § 8.6 (2016), http://www.hpcsa.co.za/Uploads/editor/UserFiles/downloads/conduct_ethics/Booklet%208%20.pdf; *see also* Ley Orgánica 2/2010, de 3 de Marzo, de Salud Sexual y Reproductiva y de la Interrupción Voluntaria del Embarazo, BOE 2010, 3514, art. 19, *translation available at* https://www.reproductiverights.org/sites/crr.civicactions.net/files/documents/Organic%20law%202-2010%20on%20SRH%20and%20voluntary%20pregnancy%20termination%20-%20English%20Translation.pdf (Spanish law stating that health-care providers may object on conscientious grounds, “provided their choice does not undermine access or the quality of care.”).

46 Zampas C, Andión-Ibañez X. Conscientious Objection to Sexual and Reproductive Health Services: International Human Rights Standards and European Law and Practice, Eur. J. Health L. 2013;19:252–53.

47 Code de la Sante Publique (Fr.), arts. L2212–8.

48 General Medical Council (UK), Good Medical Practice, 52 (2013), http://www.gmc-uk.org/Good_medical_practice___English_1215.pdf_51527435.pdf.

49 IACHR, Access to Information on Reproductive Health from a Human Rights Perspective, 94–95, 99 (2011), http://www.cidh.oas.org/pdf%20files/womenaccessinformationreproductivehealth.pdf.

50 Report of the Committee on the Elimination of Discrimination Against Women, 20th & 21st Sess., Jan 19-Feb. 5, June 7–25, 1999, ch. I, 11, U.N. Doc. A/54/38/Rev.1 (1999), http://tbinternet.ohchr.org/Treaties/CEDAW/Shared%20Documents/1_Global/INT_CEDAW_GEC_4738_E.pdf.

51 World Medical Association. WMA declaration of Oslo on therapeutic abortion. 2006. https://www.wma.net/policies-post/wma-declaration-on-therapeutic-abortion/. Accessed 31 Jan 2018; *see also* International Federation of Gynecology and Obstetrics. Ethical issues in obstetrics and gynecology. 2015:37–39. https://www.figo.org/sites/default/files/uploads/wg-publications/ethics/FIGO%20Ethical%20Issues%202015.pdf4893.pdf.

52 American College of Obstetricians and Gynecologists Committee on Ethics. Committee Opinion No. 385: The limits of conscientious refusal in reproductive medicine. 2007. https://www.acog.org/Clinical-Guidance-and-Publications/Committee-Opinions/Committee-on-Ethics/The-Limits-of-Conscientious-Refusal-in-Reproductive-Medicine (“Physicians and other health care providers have the duty to refer patients in a timely manner to other providers if they do not feel that they can in conscience provide the standard reproductive services that patients request.”). Accessed 31 Jan 2018; American Medical Association. AMA Code of Medical Ethics Opinion 1.1.7: Physician Exercise of Conscience. https://www.ama-assn.org/delivering-care/physician-exercise-conscience (“In general, physicians should refer a patient to another physician or institution to provide treatment the physician declines to offer.”). Accessed 31 Jan 2018.

53 American College of Obstetricians and Gynecologists Committee on Ethics, Committee on Health Care for Underserved Women. Committee Opinion No. 613: Increasing Access to Abortion. 2014. https://www.acog.org/Clinical-Guidance-and-Publications/Committee-Opinions/Committee-on-Health-Care-for-Underserved-Women/Increasing-Access-to-Abortion. Accessed 31 Jan 2018.

54 American College of Obstetricians and Gynecologists Committee on Ethics, Committee on Health Care for Underserved Women. Committee Opinion No. 613: Increasing Access to Abortion. 2014. https://www.acog.org/Clinical-Guidance-and-Publications/Committee-Opinions/Committee-on-Health-Care-for-Underserved-Women/Increasing-Access-to-Abortion. Accessed 31 Jan 2018.

55 Conscience Protection Act of 2017, H.R. 644, 115th Cong. (2017), https://www.congress.gov/bill/115th-congress/house-bill/644/text.

